# Tailoring microstructure of Mg–Zn–Y alloys with quasicrystal and related phases for high mechanical strength

**DOI:** 10.1088/1468-6996/15/4/044803

**Published:** 2014-07-18

**Authors:** Alok Singh

**Affiliations:** Structural Materials Unit, National Institute for Materials Science, 1-2-1 Sengen, Tsukuba 305-0047, Ibaraki, Japan

**Keywords:** complex metallic alloys, quasicrystal, magnesium alloys, microstructure, mechanical strength

## Abstract

The occurrence of a stable icosahedral (*i*-) phase, which is quasicrystalline with an icosahedral (fivefold) symmetry, on the equilibrium phase diagram of Mg–Zn–RE (RE = Y, Gd, Tb, Dy, Ho or Er) alloys opened up an interesting possibility of developing a new series of magnesium alloys for structural applications. Alloys based on the *i*-phase have been studied for the past 14 years. Ultra-high strengths combined with good ductility have been shown. Here we show two strategies for tailoring microstructures for very high strengths in Mg–Zn–Y alloys. One of them involves strengthening by a fine distribution of rod-like 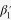 precipitates, where the matrix grain size is not critical. The alloy is solutionized at a high temperature of 480 °C to dissolve a large part of the *i*-phase, followed by a high temperature extrusion (∼430 °C) and a low temperature ageing to reprecipitate phases with fine size distribution. At first, phase transformations involved in this procedure are described. The closeness of the structure of the 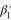 precipitates to the *i*-phase is brought out. By this procedure, tensile yield strengths of over 370 MPa are obtained in grain sizes of 20 *μ*m. In another strategy, the alloys are chill cast and then extruded at low temperatures of about 250 °C. Ultra-fine grains are produced by enhanced recrystallization due to presence of the *i*-phase. At the same time nano-sized precipitates are precipitated dynamically during extrusion from the supersaturated matrix. Ultra-high tensile strengths of up to 400 MPa are obtained in combination with ductility of 12 to 16%. Analysis of the microstructure shows that strengthening by the *i*-phase occurs by enhanced recrystallization during extrusion. It produces ultra-fine grain sizes to give very high strengths, and moderate texture for good ductility. Fine distribution of the *i*-phase and precipitates contribute to strengthening and provide microstructre stability. Ultra-high strength over a very wide range of grain sizes is thus demonstrated, by utilizing different strengthening effects.

## 1. Introduction

A stable ternary phase of composition Mg_3_Zn_6_RE exists in Mg–Zn–RE alloys (RE is a rare earth element Y, Gd, Tb, Dy, Ho or Er), which is quasicrystalline with icosahedral (*i*) symmetry [1–4]. Because of the icosahedral symmetry, it shows fivefold symmetry axes, and because of the quasiperiodicity the distance between reciprocal points along a reciprocal direction are scaled by the irrational number, the ‘Golden mean’, 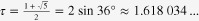. This phase forms a direct equilibrium with *α*-Mg phase, forming a two-phase field. Thus it solidifies directly as a eutectic interdendritically. It has therefore been applied as a strengthening phase for developing high strength magnesium alloys for structural applications, especially for reducing weight of automobiles. This is a very important and timely application in an urgency to control or reduce greenhouse gas emissions from automobiles, as more nations industrialize and the use of automobiles expands rapidly. Automobiles account for a major portion of use of fossil fuels. To save on precious fuel and reduce gas emissions from automobiles, weight reduction of automobiles is a major priority. Magnesium alloys are the lightest structural metals, whose use can result in major fuel savings. There have been a number of studies on the applications of magnesium alloys containing quasicrystalline phase in the past 14 years. A recent review describes the effect of icosahedral *i*-phase on the microstructure and various mechanical properties such as tensile, impact, wear and fatigue, as well as high temperature strength [5]. Here we review the main strategies to tailor microstructure of Mg–Zn–Y alloys for ultra-high strengths.

For development of high-strength alloys, microstructures must be tailored to provide for various strengthening contributions such as by grain boundary, solid solution, precipitation, dispersion and texture. There are various processing techniques such as hot rolling, extrusion, powder metallurgy, etc, which shape the alloys into sheets or bars and at the same time refine the microstructure by refining the grain size and producing a finer dispersion of the strengthening phase. Dispersion of *i*-phase in Mg–Zn–Y alloys was first shown by hot rolling by Bae *et al* [6, 7] and by extrusion by Singh *et al* [8, 9], followed by other studies [10–13]. These studies gave very encouraging results of tensile yield strengths of about 250 MPa and ductility (elongation to failure) of 20%. Further studies reported grain sizes down to about 1 *μ*m resulting in tensile and compression yield strength of about 300 MPa [14, 15]. Wrought processing such as rolling or extrusion also introduce a crystallographic texture in the alloys. In case of low symmetry metals such as magnesium, this causes anisotropy of deformation (yield asymmetry) in which yield strengths are higher in tension than in compression. In *i*-phase strengthened magnesium alloys, higher strengths could be obtained without losing much ductility, resulting in high fracture toughness [16], and where yield asymmetry was minimized [14].

In a further development, Mora *et al* [17] reported powder metallurgy processing of Mg–Zn–Y alloys to obtain sub-micron grain size and tensile yield strength of about 400 MPa. Subsequently, Singh *et al* [18, 19] showed similar strength with a much simpler technique of direct extrusion, with tensile and compressive yield strength reaching 400 MPa and elongations of over 12%. It was further demonstrated that this level of tensile strength and elongation could be retained even with alloying of yttrium as little as 0.2 at% [20].

In the following, we will review the phase transformations involving *i*-phase in Mg–Zn–Y alloys, and methods to tailor the microstructures for high strength and ductility. Three major factors are applied for strengthening: (i) grain size control, (ii) strengthening by precipitation and (iii) *i*-phase dispersion. Two major strengthening strategies are described. In one, high supersaturation of alloying elements is achieved by solutionizing at high temperatures, and very fine reprecipitation of *i*-phase and intermetallic phase precipitates is obtained. High strength (tensile yield strength 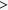 350 MPa) is obtained by very fine precipitation; the grain size is not critical. In another, very fine grain size and fine precipitates are obtained by dynamic recrystallization and dynamic precipitation by extrusion of supersaturated cast alloy to achieve tensile yield strength of over 370 MPa with elongations of 12%. Thus ultra-high strengths obtained over a wide range of grain sizes is demonstrated.

## 2. Phase equilibrium and microstructure of *i*-phase in Mg–Zn–RE alloys

**Figure 1.**
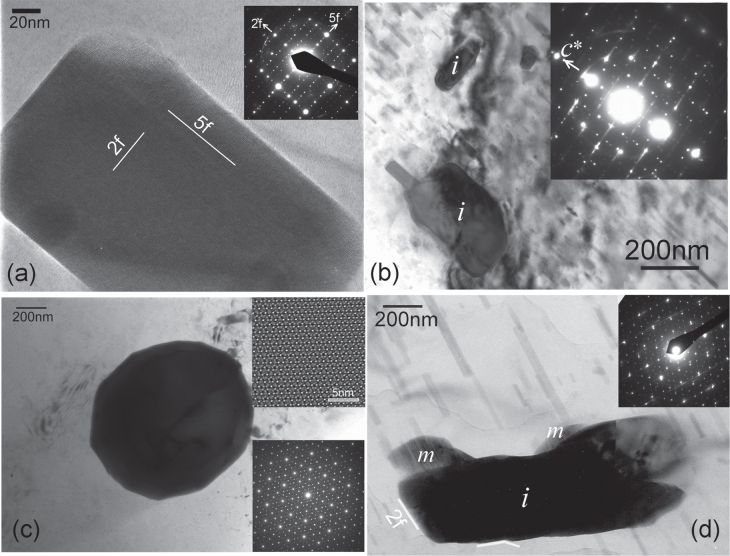
(a) An icosahedral phase particle oriented along a zone axis containing a fivefold and a twofold reciprocal vector perpendicular to each other, in a Mg-6Zn-1Ho (at%) alloy extruded at 230 °C. The inset shows the corresponding diffraction pattern. (b) Two icosahedral phase particles in a Mg-2.5Zn-0.5Y (at%) alloy solutionized at 460 °C for 15 h followed by annealing first at 250 °C and then at 400 °C. The inset shows a composite diffraction pattern from the bigger particle and the matrix. Rod-like 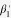 precipitates oriented along the matrix hexagonal axis are observed. (c) An icosahedral phase particle in *α*-Mg matrix, viewed along a fivefold axis, nucleated in a Mg-2.5Zn-0.5Y (at%) alloy solutionized at 460 °C for 15 h followed by annealing at 400 °C for 0.5 h. The inset shows a lattice image (top) and diffraction pattern (bottom). (d) Another icosahedral phase particle nucleated in the same alloy and oriented along a fivefold axis. The inset shows the corresponding diffraction pattern. Rod-like Mg-Zn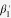 precipitates are observed in the matrix, which cause periodic streaks in the diffraction pattern.

**Figure 2.**
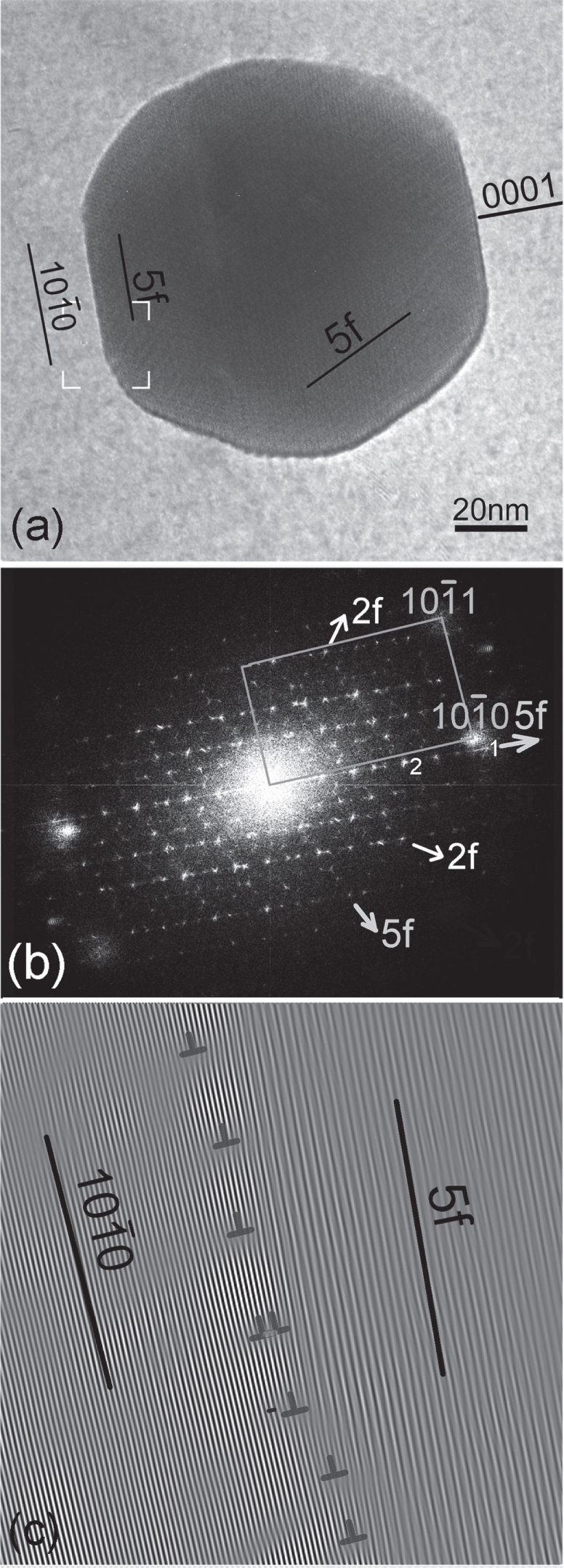
(a) An icosahedral phase particle in a Mg-4.2Zn-0.8Y (at%) alloy extruded at 400 °C, solutionized at 400 °C and aged at 200 °C, and deformed at room temperature. An FFT pattern in (b) shows it to be in a twofold zone axis orientation while the matrix is along a 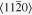 axis. (c) A Fourier filtered lattice image from a region marked in (a), showing a set of matching prismatic planes and fivefold planes corresponding to reciprocal spots {422222} and {311111} (marked ‘1’ and ‘2’, respectively, in (b)) along the fivefold reciprocal vector.

Crystallographic orientation relationship of a precipitate phase with the matrix determines its morphology. Precipitate morphology determines its effectiveness against a particular type of dislocation slip, and hence its strengthening ability. figure 1(a) shows an *i*-phase particle in a Mg-6Zn-1Ho (at%) alloy extruded at 230 °C. The inset diffraction pattern shows that it is in a 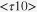 zone axis orientation in Cartesian coordinates (which is not equivalent to a fivefold axis 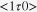, but perpendicular to it [21]). In this zone axis, a fivefold and a twofold plane are perpendicular to each other. Atomically sharp facets on these two planes are observed. When embedded inside the matrix, the *i*-phase exhibits interfaces on its fivefold and twofold planes, which have the lowest surface energy, while the matrix interfaces are mostly on basal and prismatic planes. Faceted *i*-phase particles are also observed in figure 1(b) from a Mg-2.5Zn-0.5Y (at%) alloy solutionized at a high temperature of 460 °C and then double annealed. In this micrograph the matrix is oriented close to a 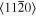 zone axis. Faceting of the particles on matrix basal and prismatic planes is observed. Figure 1(c) shows an *i*-phase particle embedded in *α*-Mg matrix in a Mg-2.5Zn-0.5Y (at%) alloy solutionized at 460 °C and aged at 400 °C. This micrograph is recorded along a fivefold zone axis (diffraction pattern inset). The round looking morphology of the particle consists of small facets. The *i*-phase in Mg–Zn–Y alloys can form many kinds of orientation relationships with the hexagonal matrix. The most common of these has an icosahedral twofold symmetry axis parallel to the hexagonal axis of the matrix 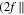
[0001]), with two possible common symmetrical variants and two other occasional variants [22–24]. In these orientation relationships, the *i*-phase exhibits a plate-like morphology on the basal plane of the hexagonal matrix, such as in figure 1(b) because the matching icosahedral twofold and the hexagonal planes make a low energy interface. In another orientation relationship, a fivefold plane matches with the hexagonal plane of the matrix. In yet another orientation relationship, an axis of the kind 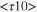 is parallel to the hexagonal axis of the matrix. In this case, the morphology of the *i*-phase is more rounded, such as shown in figure 2. Tilting experiments showed that the particle in figure 1(c) has an orientation relationship 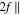
[0001], but not symmetrically oriented, called OR5 [23]. Due to this asymmetrical orientation, the particle assumes many facets, some of which are on the high density planes of the *i*-phase and the others on those of the matrix. Figure 1(d) shows another *i*-phase particle oriented along a fivefold axis. The streaks in the diffraction pattern are from the rod-like 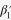 precipitates in the matrix, aligned along the matrix hexagonal axis. The symmetry of these streaks and spacing with respect to the *i*-phase diffraction brings out the crystallographic match between the phases. The surface of the *i*-phase particle appears to be rough, especially the lower edge. On a closer look, small facets on twofold planes corresponding to those in the diffraction pattern can be observed. Two instances are highlighted by white lines. Such small facets are likely to form due to match with the matrix plane, depending on the orientation relationship. Some outcrops on the particle are the monoclinic phase Mg_4_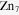 marked ‘*m*’.

Although the interfaces between the *i*-phase and the matrix are faceted, it is important to know if these are matching/epitaxial interfaces, since one lattice is periodic and another quasiperiodic. The nature of interfaces can influence the mechanical properties. Occurrence of coincidence of reciprocal spots indicates that a coherency may indeed occur at the interfaces. The probability of such occurrence is increased substantially because of the quasiperiodicity of one of the lattices, because a quasiperiodic reciprocal space has high density of spots and because the quasiperiodicity generates a wide range of interplanar spacings, which are not just multiples of each other. This creates a high probability of coincidence with a spot in a periodic lattice [25]. This matching in reciprocal space is a manifestation of epitaxial relationship in real space [26]. A quasiperiodic lattice can form epitaxial interfaces with a variety of periodic lattices which are otherwise incommensurate with each other. A quasiperiodic lattice can thus act as a ‘glue’ between two periodic lattices incommensurate with each other.

**Figure 3.**
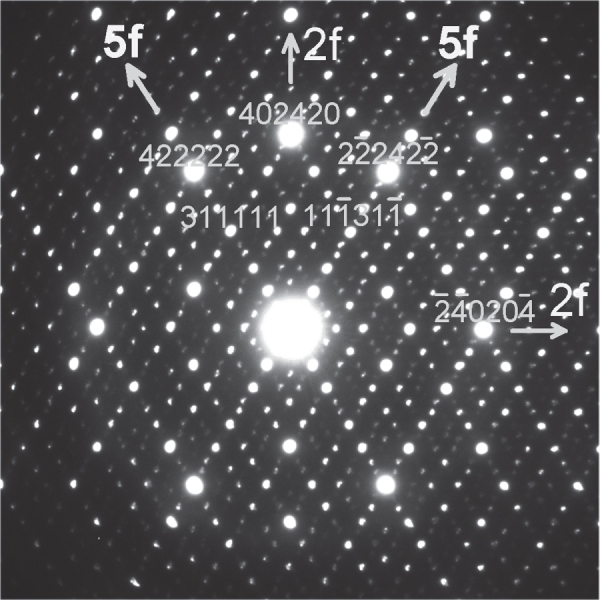
A typical twofold symmetry diffraction pattern from an icosahedral phase in a Mg–Zn–Y alloy. Fivefold and twofold reciprocal vectors are marked.

In the reciprocal space of an *i*-phase, two very prominent reciprocal spots or peaks occur. The most intense one is indexed {211111} following Elserʼs six integer indices [27]. It occurs along the fivefold reciprocal vectors. The other, with about 80% intensity of this, is {221001} which occurs along twofold reciprocal directions. These are shown in a typical twofold symmetry diffraction pattern in figure 3. Since this *i*-phase is face-centered F type ordered in the hyper space, these indices are doubled [28]. In case of Mg–Zn–Y *i*-phase, the interplanar spacing from these two spots {422222} and {442002} are 2.43 Å and 2.35 Å, respectively. Because of the highest intensity of these two peaks, the fivefold and twofold planes can be taken as the highest density or the closest packed planes in the *i*-phase. In this diffraction pattern, *τ*-inflated (i.e., quasiperiodically related) {422222} peaks {311111} are also indexed. The high intensity diffraction peaks in the magnesium reciprocal lattice are {101} (2.54 Å), {002} (2.60 Å) and {100} (2.78 Å). The first two of these are especially close in interplanar spacing to the close packed *i*-phase lattice planes. Other prominent magnesium lattice planes such as {103} with spacing of 1.47 Å can also be matched with *i*-phase twofold planes, for example, with a lattice spot at 2.43/*τ* (∼1.45 Å) {332002}. In this way, thus, a complete epitaxy on all facets can be achieved in all orientation relationships. In accordance with the closest packed planes, the interfaces between the *i*-phase and the magnesium matrix are on *i*-phase fivefold and twofold planes and magnesium basal and prismatic planes.

**Figure 4.**
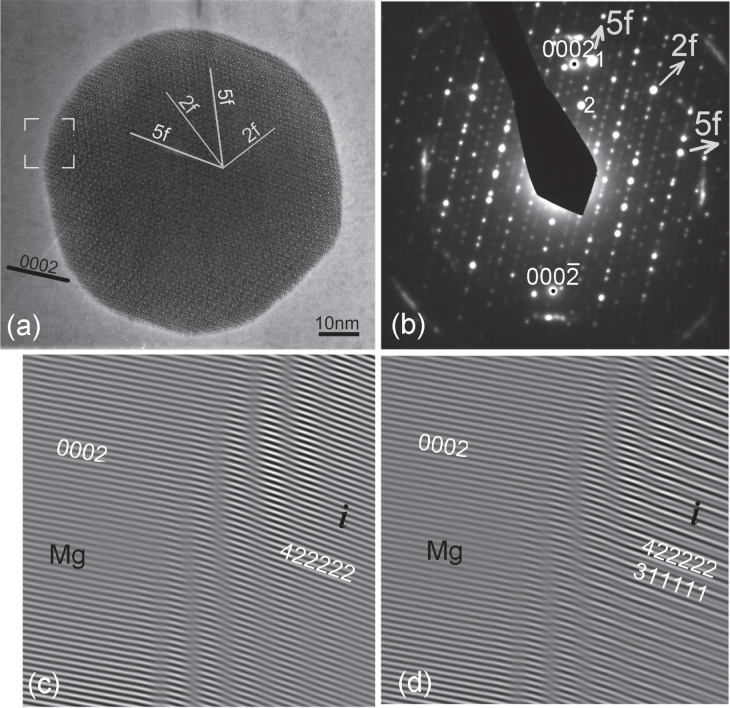
(a) An *i*-phase particle oriented along a twofold axis in a Mg-6Zn-1Ho (at%) alloy extruded at 230 °C. A diffraction pattern is shown in (b). Matrix (0002) diffraction spots are also observed in the diffraction pattern. Two prominent diffraction spots {422222} and {311111} along a fivefold vector are marked 1 and 2, respectively. Two Fourier filtered images from an area marked in (a) are shown in (c) and (d). In (c) the matrix (0002) and matching *i*-phase {422222} spot is used for inverse FFT, while in (d) the matrix (0001) and two fivefold vector spots {422222} and {311111} are used for inverse FFT.

Figure 2 shows an *i*-phase particle in a Mg–Zn–Y alloy, extruded and heat treated. The fast Fourier transform (FFT) pattern in (b) shows that a fivefold reciprocal vector has a close match with a matrix prismatic plane vector. There is no match of the matrix basal plane with any prominent plane of the *i*-phase. The particle shows sharp facets on fivefold planes and also on twofold planes. A Fourier filtered lattice image of the interface in the area marked in (a) is shown in (c). The slight mismatch in tilt between the matrix prismatic planes and the fivefold plane are clearly accommodated by the array of dislocations marked in the figure. A well matched interface is thus demonstrated. Another example is shown in figure 4, in which also the *i*-phase particle is oriented along a twofold zone axis. Fourier filtered lattice images from an area marked in (a) are shown in (c) and (d). Figure 4(c) shows an inverse Fourier transform from matrix (0001) and the matching {422222} reciprocal spots. A continuity of the lattice fringes across the interface is observed. Whatever the structural interpretation of these lattice fringes, it clearly shows an epitaxial relationship. A waviness at the interface can be ascribed to overlapping of the crystals. In figure 4(d), two *i*-phase reciprocal spots along the fivefold vector {422222} and {311111} are used for the inverse Fourier transform, which make a quasiperiodic lattice. The position of the interface is seen more clearly in this case, and a continuity of the lattice is observed.

The ternary cubic phase W-Mg_2_Zn_3_Y_3_ is a competing phase to the *i*-phase in Mg–Zn–Y alloys. It forms at temperatures higher than that of the *i*-phase. At high temperatures of 560° the *i*-phase can transform to the W-phase. The W phase shows a unique orientation relationship with the Mg-matrix, a Burgers orientation relationship 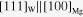 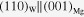. The crystallographic orientation relationship between the W-phase and the *i*-phase is given as 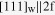 {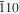}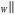 2f [29]. The interplanar spacing 2.41 Å of the {111} plane is close to the interplanar spacings corresponding to the most intense diffraction peaks of the *i*-phase {422222} and {442002}. Other corresponding planes also have matching interplanar spacings. The W phase can react with the matrix to form *i*-phase on the interface [30]. The *i*-phase grows on the W-phase with either a fivefold axis or a twofold axis occurs along the 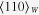 axis which is parallel to the hexagonal axis of the matrix. This results in two orientation relations between the *i*-phase and the matrix:

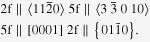



**Figure 5.**
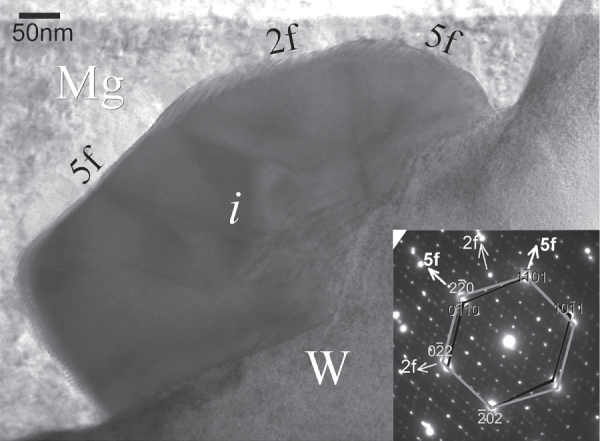
A multibeam transmission electron microscopy (TEM) micrograph showing icosahedral phase grown on W-phase in *α*-Mg matrix of Mg-2.5Zn-0.5Y (at%) alloy solutionized at 460 °C followed by annealing at 400 °C. The composite electron diffraction pattern (inset) gives the zone axis orientation of the three phases, the icosahedral phase in the twofold zone axis, the W-phase in the [111] zone axis and the *α*-Mg phase in the 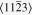 zone axis.

Figure 5 shows a situation in which 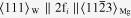 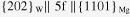. The interfaces between *i*-phase and the matrix are faceted. Tilting the sample showed that these facets are on fivefold and twofold planes of the *i*-phase and prismatic and basal planes of the matrix [30]. Interfaces between the *i*-phase and W-phase in this micrograph appear to be faceted on twofold and fivefold *i*-phase planes, but did not appear faceted when observed in other orientations, leading to the conclusion that the interfacial energy between these two phases is low, due to some similarity in structure [29].

The *i*-phase was reported to form a peritectic reaction with a phase of composition 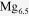
Zn_77_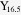 [31], presumably the same as reported to be (Zn,Mg)_5_Y [32, 33], later identified as hexagonal (H) [3] with composition Mg_5_Zn_79_Dy_16_ [34]. On solutionizing our sample of a Mg-2.5at%Zn-0.5at%Y alloy at 460 °C for 15 h, the *i*-phase transforms to a hexagonal phase with lattice parameters *a* = 9.18 and *c* = 9.5 Å, and composition Mg_24_Zn_59_Y_17_, which can also be written as (Mg,Zn)_5_Y [35]. It forms an orientation relationship 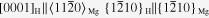 with the matrix and 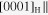 2f 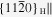 2f with the *i*-phase.

## 3. Mg–Zn binary phases

The RE elements in Mg–Zn–RE alloy tend to diffuse to the grain boundaries, forming ternary phases such as the *i*-phase, while inside the grains or the matrix, the phase equilibrium is essentially Mg–Zn binary [36]. Precipitation of binary phases occurs in the matrix. Several binary phases are reported in the Mg–Zn system [37]. These include Laves hexagonal phase MgZn_2_ (P6_3_/mmc *a* = 5.22 Å, *c* = 8.57 Å), monoclinic phase Mg_4_Zn_7_ (C2/m *a* = 25.96 Å, *b* = 5.24 Å, *c* = 14.28 Å, 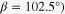, orthorhombic (pseudo-cubic) Mg_7_Zn_3_ (Mg_51_Zn_20_) (Immm *a* = 14.08 Å, *b* = 14.49 Å, *c* = 14.02 Å), triclinic Mg_2_Zn_3_ (*a* = 17.24 Å, *b* = 14.45 Å, *c* = 5.2 Å, *α* = 96°, *β* = 89°, *γ* = 138°), and rhombohedral MgZn (hexagonal *a* = 25.58 Å, *c* = 18.15 Å). The lattices of these phases are related to each other through a lattice parameter which is related to the *c* parameter of the matrix *α*-Mg. Some of these relationships are [38]

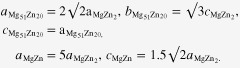



**Figure 6.**
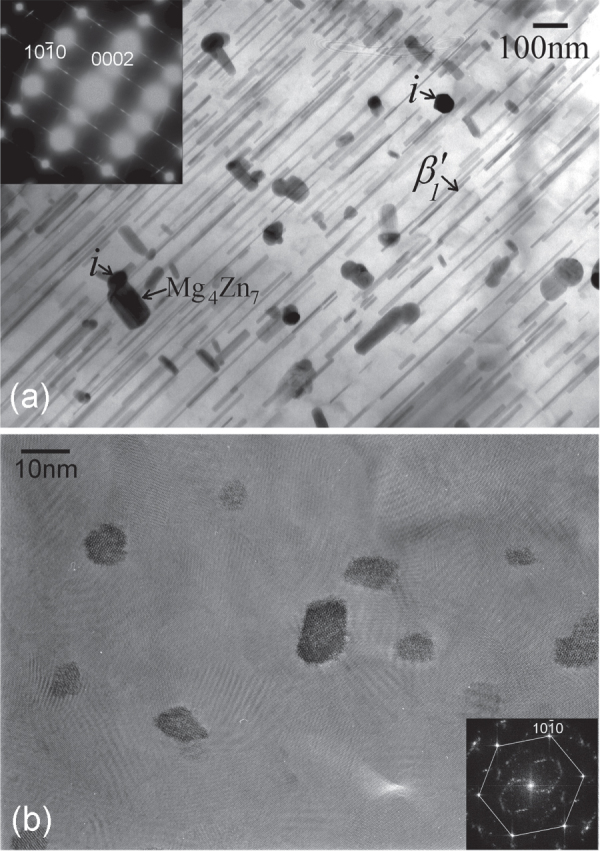
Precipitates in a Mg-6at%Zn-1at%Y alloy solutionized at 440 °C, extruded at 380 °C and aged at 150 °C. (a) A bright field micrograph along a 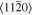 zone axis showing rod-like 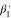 precipitates, *i*-phase particles and Mg_4_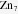 phase growing on the *i*-phase. (b) A lattice image along the hexagonal axis of a matrix grain showing 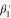 precipitate rods viewed head on. The inset shows the corresponding diffraction pattern.

These phases are related to each other and also related to the *i*-phase through structural blocks of icosahedrally coordinated atoms. The most important strengthening precipitate which occurs in Mg–Zn and related alloys is rod-like 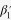, which grows parallel to the hexagonal axis of the *α*-Mg matrix, and hence is effective against basal slip of dislocations, the most prominent deformation mode in magnesium alloys. These precipitates can be observed in figure 1(b) and figure 6. For a long time, its structure has been believed to be based on the 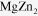 phase [36], until shown to be based on the Mg_4_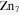 phase instead [39, 40].

**Figure 7.**
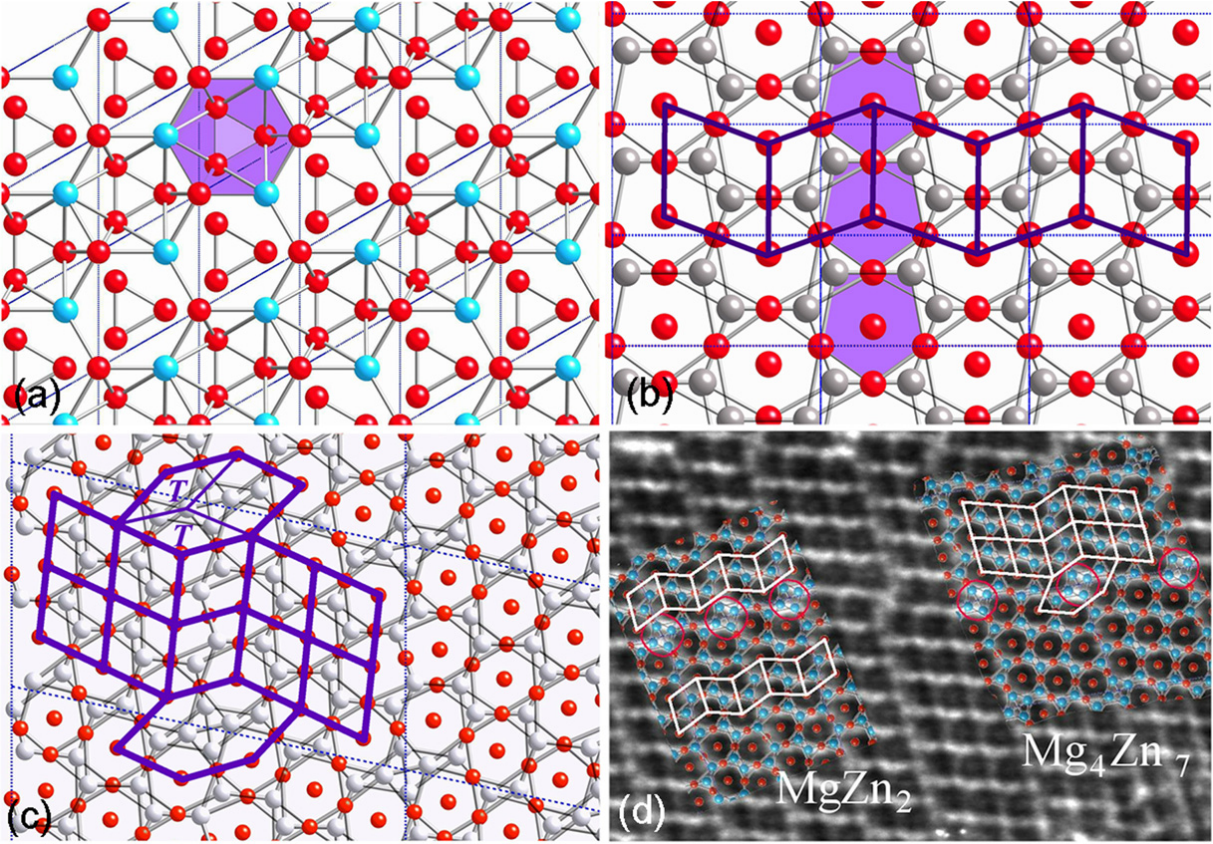
Layered structure of the 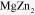 and Mg_4_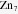 phases and an intermixing of the two. (a), (b) The structure of the 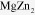 phase represented as stacking of layers of Mg and Zn atoms along [0001] axis (a) and a 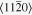 axis (b). An icosahedral unit in (a) is highlighted. Pentagonal arrangements, which make icosahedral units, are observed in (b). Centers of these pentagons are arranged as rhombuses. (c) Structure of the Mg_4_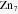 phase viewed along the unique monoclinic axis [010]. It is represented as stacking of layers with pentagonal coordinations, making icosahedra. The coordinations are similar to those in the 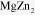 structure along 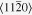 axis in (b), except for a hexagonal unit in which zinc atoms are concentrated. The hexagonal unit can be further decomposed into a thick and two thin tiles, as shown in the upper hexagon. (d) A high resolution TEM micrograph of a rod-shaped 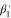 precipitates viewed along its long axis. Structure of 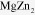 phase on the left and Mg_4_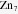 phase on the right can be identified, as outlined. Regions of zinc concentration are marked with circles.

The structure of 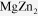 phase can be considered as two layers stacked along its hexagonal axis [41], as shown in figure 7. A layer of Zn and Mg atoms and another of Zn atoms, when stacked, make icosahedral coordinations. Along the hexagonal axis of the lattice, these icosahedra are oriented along their threefold axes, as observed in figure 7(a). When viewed along a 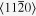 axis, these icosahedra are oriented along a fivefold axis (figure 7(b)). Joining the centers of these icosahedra make a tiling of ‘fat’ rhombuses (with acute angle of 72°) of Penrose tiling, as outlined in figure 7(b). The structure of the Mg_4_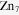 phase is a stacking of layers along its unique axis [010] [42]. Four layers form a unit cell. Two of these are primary layers, which have pentagonal and triangular arrangement of atoms. These two layers are rotated with respect to each other by 180°. Secondary layers in between are formed of zinc atoms at positions corresponding to the centers of pentagons in the primary layers, so that the stacking forms interlocked icosahedra [43], shown in figure 7(c). Thus the structures of these two phases are similar. Connecting the centers of icosahedra in Mg_4_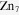 structure also makes thick rhombuses, as well as a hexagonal unit (figure 7(c)). This hexagonal unit is deficient in zinc atoms, which is responsible for the difference in stoichiometry of the two phases. The hexagonal unit can be further decomposed into two thin (acute angle of 36°) and one thick rhombuses.

Figure 6(b) is a view of the cross-section of 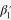, along the hexagonal axis of the matrix *α*-Mg. If these precipitates have a structure of Mg_4_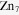 phase, they are along the 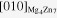 zone axis, and if these precipitates have a structure of 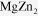 phase, they are oriented along the 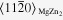 zone axis. Indeed both these phases can coexist in the 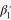 precipitates, because of the similarity of their structures [44–46] as shown in figure 7(d), with crystallographic axial relationship 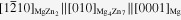. Maintaining this axial relationship, the Mg_4_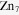 phase can make one of two planar matches 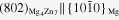 or 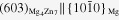. In turn, the 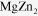 phase can form two equivalent orientation relationships with the Mg_4_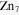 phase 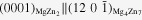 or 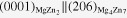. Since the structure of the 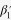 precipitates is partly or predominantly Mg_4_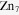 phase, its morphology in *α*-Mg matrix is rod-like, parallel to the matrix hexagonal axis, due to its growth direction and the lattice match with the matrix. This is very helpful for strengthening of the alloys, as will be shown in subsection 3.1. After a transformation to 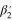 precipitates, whose structure is completely 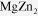 phase with plate-like morphology in the matrix basal plane, the strength of the alloy is reduced [47].

**Figure 8.**
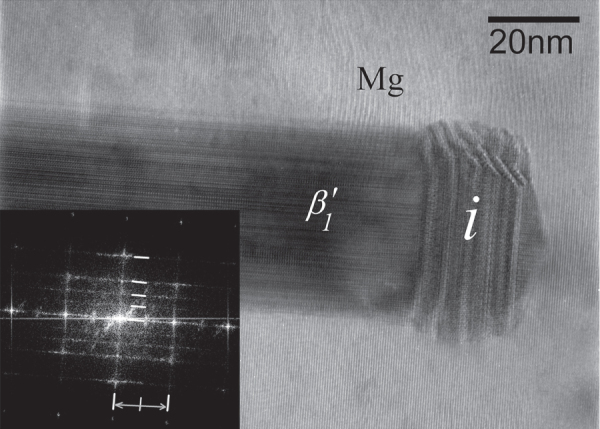
Intergrowth of icosahedral (*i*) and a 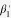 precipitate in *α*-Mg matrix in Mg-2.5Zn-0.5Y (at%) solutionized at 460 °C and a double annealed at 250 °C and 400 °C. The inset shows an FFT, in which horizontal streaks are arranged quasiperiodically while the vertical streaks have the periodicity of basal planes of *α*-Mg phase and 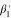 precipitated phases.

Since the structures of 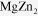 and Mg_4_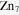 phases consist of icosahedral coordinations, they must have structural similarity to the *i*-phase. Compositionally, the *i*-phase contains the ternary element yttrium or a rare earth element. It is observed in figure 6(a) that Mg_4_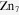 phase can nucleate and grow on *i*-phase particles, as a tail-like feature. They grow with orientation relationship [010]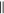 5f on the *i*-phase. Conversely, *i*-phase can also grow on 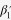 precipitates. Figure 8 is an example from an alloy solutionized at 460 °C and then double annealed. This micrograph is recorded along a 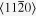 zone axis of the matrix. Annealing at 250 °C resulted in precipitation of 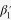 precipitates. However, this temperature was not high enough to diffuse yttrium and to nucleate *i*-phase. It was then annealed at 400 °C, after which formation of *i*-phase was observed. In this instance, nucleation of *i*-phase has occurred on the tip of a 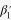 precipitate. In the inset FFT, horizontal lines (streaks) are stacked quasiperiodically. From the base line their distance follows a Fibonacci sequence of 3, 5, 8 and 13. The horizontal periodicity (of vertical streaks) is related to the basal planes of the matrix and (010) planes of Mg_4_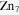 phase. The *i*-phase has grown in layers, possibly related to the diffusion rate of yttrium.

### 3.1. Strengthening by β_1_^′^ precipitates

Rod-shaped 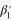 precipitates in Mg–Zn alloys are the primary source of strengthening, and basis of the ZK series of commercial alloys. Their alignment along the hexagonal axis effectively strengthens against basal slip, the primary mode of deformation in magnesium alloys. Rosalie *et al* [48, 49] have quantitatively determined the effect of distribution parameters of these precipitates on the strength and ductility of the alloys. The size and the distribution parameters (aspect ratio and number density at a constant volume fraction) has been related to the strength and ductility. Following the Orowan strengthening mechanism, the increase in yield strength (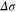) from a given volume fraction of non-shearable precipitates is given as [50]

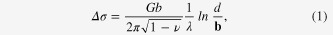
where *ν* is Poissonʼs ratio, *G* shear modulus in GPa and 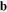 is the Burgers vector for 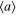 dislocations in magnesium. *d* is the diameter and *λ* is the spacing of the precipitates (cross-section) on the basal planes. For rod-shaped precipitates, these two parameters are related by [50]

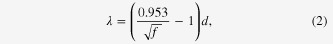
where *f* is the volume fraction of the precipitates. Thus, for a particular volume fraction of precipitates, a reduction in the size of the precipitates will reduce the precipitate spacing and increase the strength. This refinement of precipitates is usually achieved by micro-alloying. It can also be achieved by increasing heterogeneous nucleation sites such as dislocations by straining the alloy (T8 treatment), as followed by Rosalie *et al* [48, 49]. For a Mg-3at%Zn alloy, the increment in strength as a function of precipitate spacing 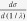 is shown to be 3100 MPa nm [48]. With increase in strength, however, the ductility is decreased drastically, which is also a function of *λ*. For an average volume fraction 3.5% of precipitates, with *d* = 14 nm the yield strength (in peak aged condition after solutionizing, T6 treatment) was obtained to be 273 MPa (elongation 17%), and with *d* = 9 nm (in T8 condition), the yield strength was 305 MPa (elongation 6%). Yield strength without precipitates (in solutionized condition) was obtained to be 143 MPa (24% elongation) [48].


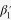
 precipitation was also studied in a ternary Mg-3.0Zn-0.5Y (at%) alloy [49]. Since ternary *i*-phase forms in this alloy, the volume fraction of precipitates will be less than the Mg-3Zn (at%) binary alloy. In solutionized and peak aged (T6) condition, the volume fraction of precipitates was measured to be 0.5%. After straining and ageing (T8), not only was the precipitate size and distribution refined, but the volume fraction of the precipitates increased to about 2.3%. Thus the straining changes the phase equilibrium in the alloy, possibly by diffusion of yttrium through dislocations.

**Figure 9.**
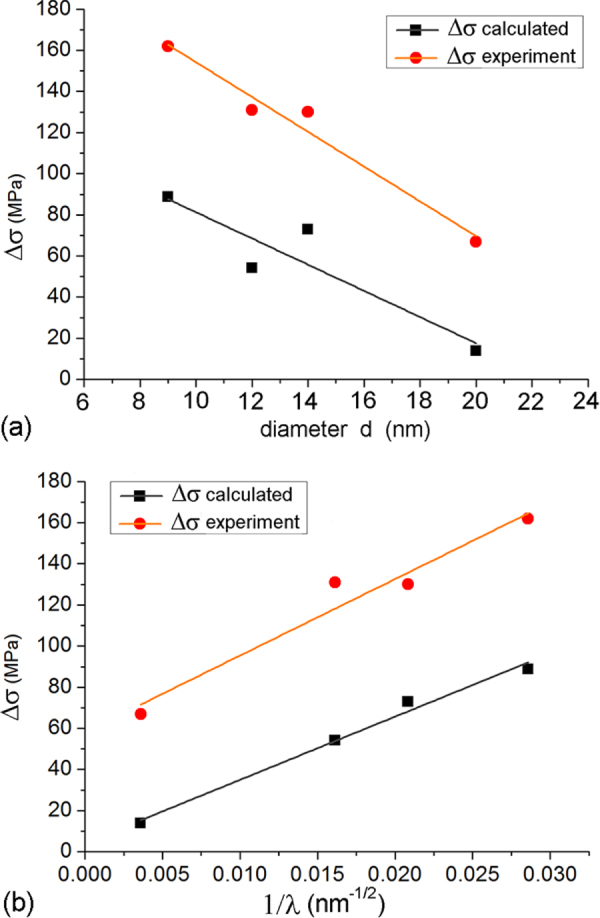
Calculated and experimental increments in yield strength 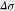 plotted against experimentally determined values of 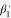 precipitate diameters *d* and precipitate spacing on the basal planes in T6 and T8 treated (a) Mg–Zn and (b) Mg–Zn–Y alloys given in [48, 49].

By using experimentally determined values of *d* and *λ* in [48, 49], 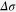 were calculated using equation (1) for Mg–Zn and Mg–Zn–Y alloys in T6 and T8 conditions, and plotted in figure 9. Experimental 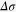 were taken to be the yield strength subtracted by the yield strength in solutionized condition (before precipitation). It is observed that all the points follow the same linear relationship, and that there is good correspondence between the calculated and experimental plots, although they differ by a scaling factor. Thus precipitation and its distribution is a major strengthening factor in these alloys.

## 4. Tailoring of microstructure of Mg–Zn–Y alloys

Of the common strengthening strategies, grain reduction is especially effective in the case of magnesium alloys. It is commonly achieved through wrought processing such as rolling and extrusion which, at the same time, disperses the icosahedral and other ternary phases more finely, from the coarse dendritic solidification structure. The dispersion is, however, not uniform and the *i*-phase particles range in size from several microns to about 500 nm in size. Another important consequence of the wrought processing is the introduction of a texture. In this texture, also known as basal texture, the grains align themselves with their basal planes along the rolling or the extrusion direction. This introduces a deformation anisotropy in the alloy. During tensile testing along the rolling or extrusion direction, orientation of the grains is unfavorable for activation of basal slip or twinning. Therefore deformation is difficult and the tensile strength is high. Compression along the same axis, however, favors easy twinning. The yield strengths in tension are therefore higher than in compression.

Texture also has a profound effect on ductility. When the basal texture is strong, basal slip is difficult. Therefore the yield strength is high, but the ductility is low. When the texture is weaker, the strength is lower but the ductility is higher. Therefore the texture can be modified to balance strength with ductility. It will be shown that presence of the *i*-phase helps in attaining finer grain sizes and weaker textures by wrought processing.

### 4.1. Processing for precipitation dominated strengthening

**Figure 10.**
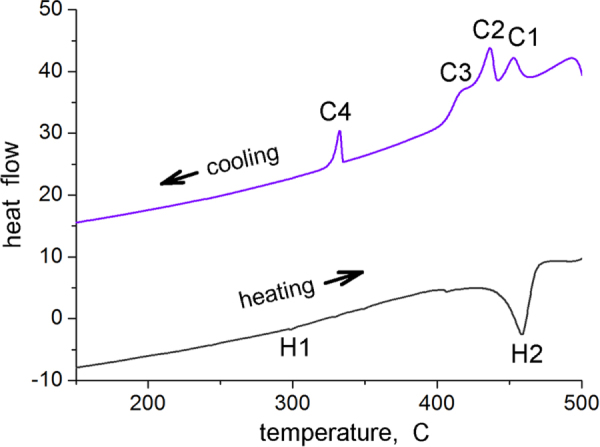
DSC traces of heating and cooling runs of Mg-6Zn-1Y (at%) alloy after solutionizing at 480 °C for 24 h.

An approach to finer dispersion of *i*-phase in the alloy is dissolution of the *i*-phase by high temperature solutionizing and re-precipitation. In this way, the eutectic solidification structure is first broken, followed by extrusion. Nucleation of the *i*-phase occurs on new grain boundaries formed by extrusion. This procedure follows the phase transformations described in section 2. The extrusion temperature is kept high for easy nucleation of the *i*-phase. Figure 10 shows differential scanning calorimetry (DSC) plots for a Mg-6at%Zn-1at%Y alloy solutionized at a temperature of 480 °C for 24 h after solidification. On heating, an endothermic peak occurs at H2 with a maximum height at about 460 °C, which corresponds to dissolution of *i*-phase. On cooling from 500 °C, a set of three peaks (C1 to C3) from about 460 °C to about 400 °C occur. These can be correlated to nucleation of the *i*-phase. A set of three peaks indicates three distinct nucleation sites. The first peak at C1 shows an undercooling of only about 6° with respect to peak H2, peak C2 about 22° and peak C3 about 42°. Nucleation sites can be grain boundaries, over existing ternary phases such as 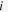 and W phases, and on precipitates and dislocations in the matrix. The exothermic peak at C4 (332 °C) is for the nucleation of 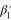 precipitates.

**Figure 11.**
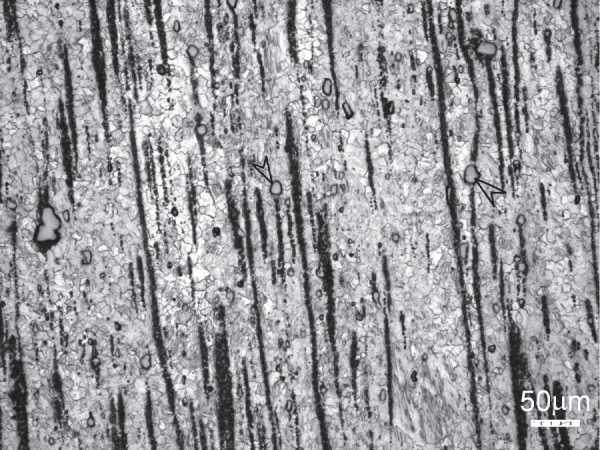
Microstructure of a Mg-6at%Zn-1at%Y chill cast alloy, solutionized at 480 °C and extruded at 440 °C. Two of the large particles of cubic W-phase are marked with arrowheads.

**Figure 12.**
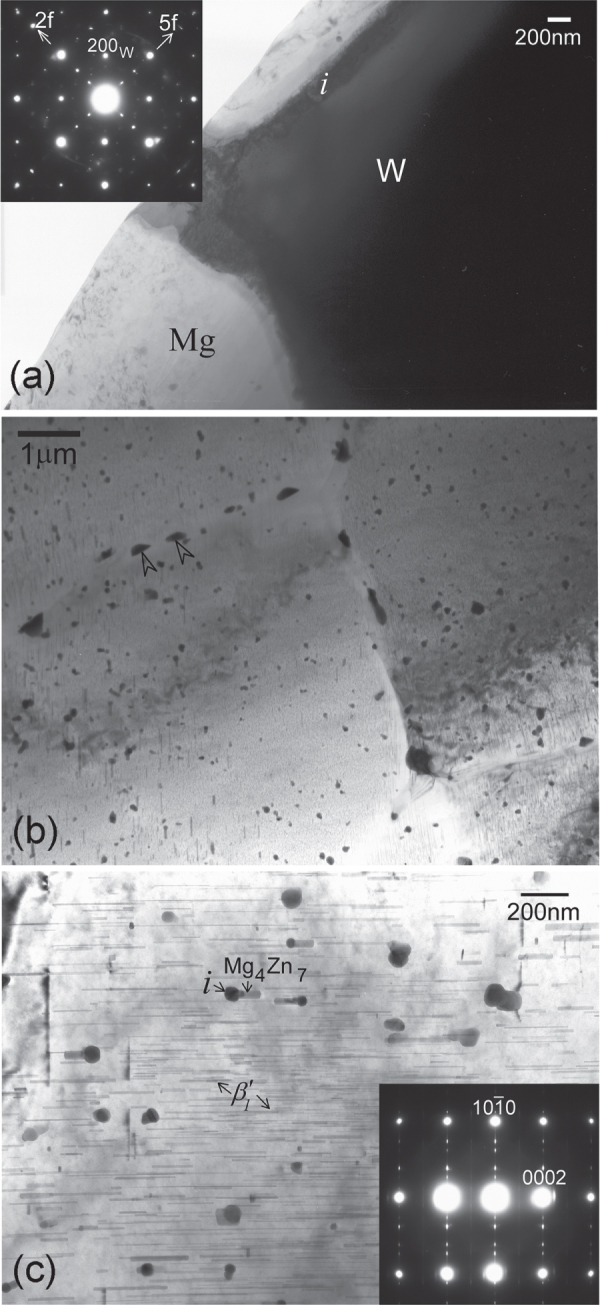
Microstructure of Mg-6at%Zn-1at%Y chill cast alloy, solutionized and extruded at 440 °C. (a) W-phase with *i*-phase at the interface. In the inset diffraction pattern, *i*-phase and W-phase diffraction spots are observed. The W-phase is in the [100] zone axis orientation. Icosahedral twofold (2f) and fivefold (5f) reciprocal directions are marked. (b) Microstructure after ageing at 150 °C for 48 h; *i*-phase particles at grain boundaries are marked with arrowheads. (c) 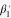 precipitates and *i*-phase particles are observed in a grain oriented along a 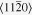 zone axis.

**Figure 13.**
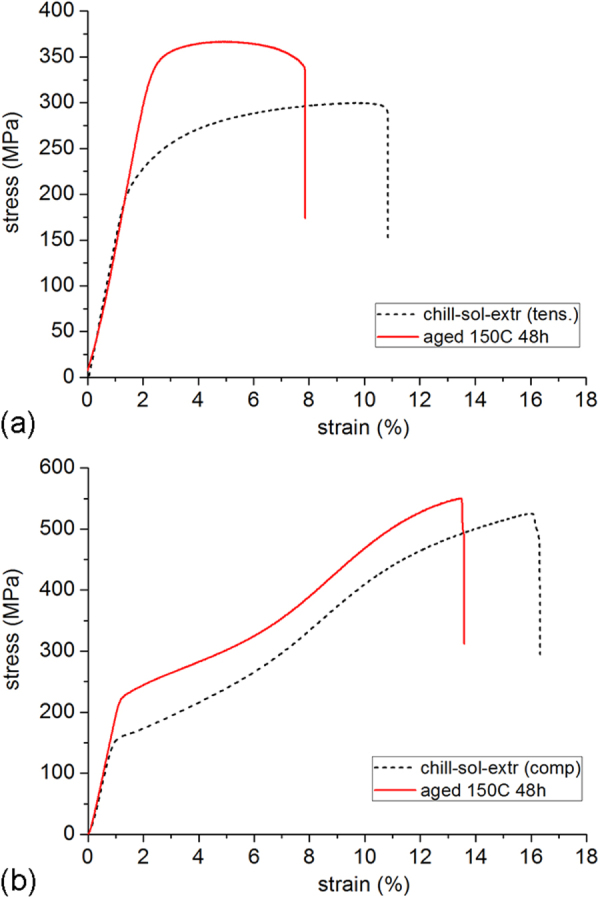
Stress–strain curves in (a) tension and (b) compression of chill-cast, solutionized and extruded Mg-6at%Zn-1at%Y alloy in as-extruded condition and after ageing at 150 °C for 48 h.

Examination of the solutionized microstructure showed dissolution of *i*- phase and partly transformation to H-phase. High temperature phases H and cubic W formed by transformation from the *i*-phase. In addition, free yttrium was also detected, as a result of dissolution of the *i*-phase [51]. The cooling curve of DSC shows three close exothermic peaks in the temperature range of 470° to 410 °C (figure 10). Extrusion was carried out at 430 °C. Figure 11 shows as-extruded microstructure. A microstructure of grains of about 20 *μ*m in size is observed. Strings of dark particles are the *i*-phase dispersed along the extrusion direction. TEM micrographs in figure 12 show *i*-phase particles of about 200 nm in size along the grain boundaries. A dispersion of *i*-phase particles of about 50–100 nm in size also occurred in the grains. This alloy was tested for strength in tension and compression. Figure 13 shows the stress–strain curves of tensile and compression tests. The tensile and compressive yield strengths were about 210 and 170 MPa, respectively. Elongation in tension was nearly 12%, while in compression was about 16%.

Ageing at a low temperature of 150 °C resulted in very fine precipitation of rod-shaped 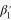 precipitates from the highly supersaturated matrix. Figure 12(c) shows precipitation after ageing at 150 °C for 24 h. These precipitates had average length of about 25 nm and width about 7 nm. By using equation (1), this gives a strength increment 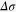 of about 150 MPa. The strength of the alloy increased dramatically, as observed in the tensile and compression graphs of figure 13. Tensile yield strength was now 365 MPa, a 58% increase, and compression yield strength 268 MPa, a 64% increase. The tensile elongation is, however, now limited to 8%. This is believed to be caused by large W-phase particles formed during solutionizing treatment, acting as inclusions. Figure 11 shows some large gray particles encased in black shells; two of these are pointed out by arrowheads. These are identified as W-phase with *i*-phase growing over them. One such instance identified by TEM is shown in figure 12(a). The composite diffraction pattern inset gives the orientation relationship between W- and *i*-phase described in section 2 and figure 5. However, it has also been shown that a lowered ductility is a result of the refinement of precipitation and the consequent rise in strength, section 3.1 [48, 49].

### 4.2. Ultra-fine grain size by direct extrusion

**Figure 14.**
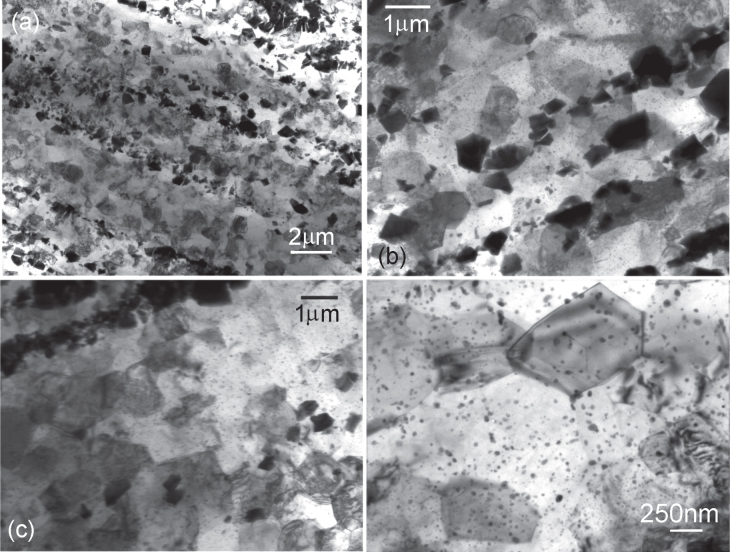
Bright field TEM micrographs showing microstructures of extruded Mg–Zn–Y alloys produced by chill casting. Fine micron level grain sizes are observed. The black particles are the dispersed *i*-phase. (a) Mg-6Zn-1Y alloy extruded at 250 °C. (b)–(d) Mg-3Zn-0.5Y alloy extruded at (b) 300 °C, (c) 250 °C and (d) 300 °C. Fine nano-sized precipitates in the matrix are clearly observed in the higher magnification micrograph of (d).

Another approach to finer dispersion in an alloy by wrought processing is through a better casting structure, in which the dendrite size is finer. Using this approach, an alloy of the same composition as above (section 4.1), Mg-6at%Zn-1at%Y, and another of composition Mg-3at%Zn-0.5at%Y were cast in a chill-cast mold made of thick steel. These cast alloys were then directly extruded. Each alloy was extruded at three different temperatures to obtain various grain sizes. Representative microstructures of the alloys are shown in figure 14. Black particles, about a few hundred nanometers to a micron in size, are the *i*-phase dispersed in the matrix along the extrusion direction. Extrusion at temperatures lower than 300 °C resulted in matrix grain sizes of about a micron. In all the extruded alloys fine precipitation of 10–20 nm in size occurred. This is more clearly observed in figure 14(d). These are believed to have dynamically precipitated during extrusion from a supersaturated matrix, and comprise *i*-phase or monoclinic Mg_4_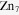 phase. Average grain sizes obtained by extrusion are listed in table 1.

**Table 1.** Extrusion temperatures and the corresponding grain size, tensile and compressive yield strengths (YS), and tensile elongations of the two alloys Mg-6Zn-1Y and Mg-3Zn-0.5Y (at%) which were chill cast and extruded, and then aged.

Alloy	Extrusion	Grain	Tensile	Tensile	Compressive
	temp.,°C	size *μ*m	YS, MPa	elong., %	YS, MPa
Mg-6Zn-1Y	390	5.14	268.3	12.9	252.9
	300	1.13	340.5	14.8	342.3
	250	0.98	386.3	16.0	354.6
Mg-6Zn-1Y	390	6.00	288.7	17.3	258.1
aged	300	2.32	346.4	14.0	326.2
	250	1.61	399.9	10.5	377.6
Mg-3Zn-0.5Y	350	4.33	262.0	18.3	299.4
	300	2.05	348.2	16.3	274.7
	260	1.39	373.3	13.4	316.4

**Figure 15.**
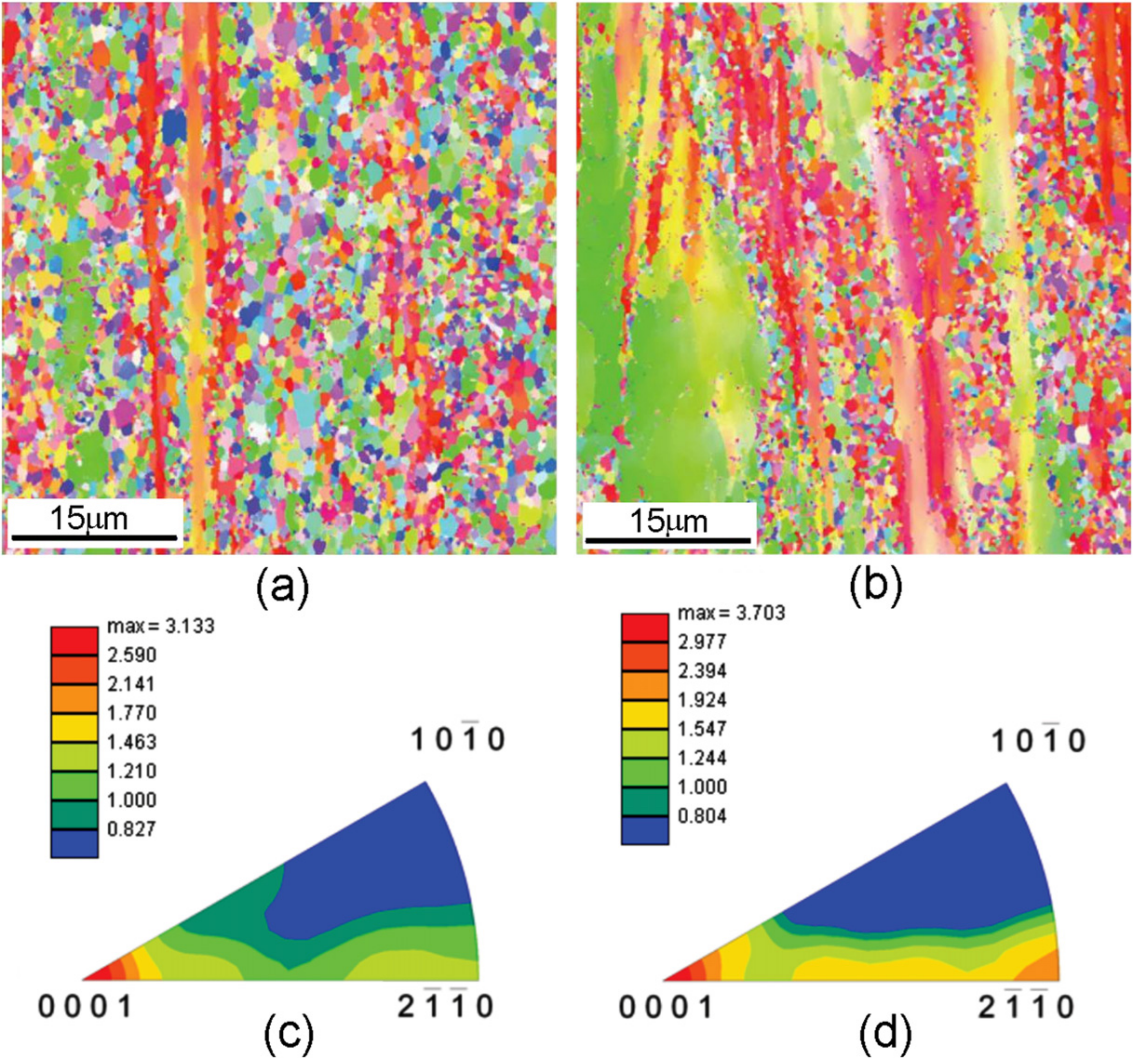
(a), (b) Inverse pole figure maps and (c), (d) the corresponding inverse pole figures obtained by EBSD for the alloys Mg-6Zn-1Y extruded at 300 °C (a), (c) and Mg-3Zn-0.5Y alloy extruded at 260 °C (b), (d). Reproduced in part from [52] with permission from Elsevier.

Crystallographic orientation data over a large area were obtained by electron backscattered diffraction (EBSD) technique using orientation imaging microscopy (OIM) software [52]. Representative data are shown in figure 15 for two alloys Mg-6Zn-1Y and Mg-3Zn-0.5Y extruded at 300 °C and 260 °C, respectively. Grain orientations are represented by colors. Both of these alloys have average grain sizes in the range of 1.1 to 1.4 *μ*m. However, some clear differences between these two alloys are observed. The grains in (a) have more contrasting colors, which is representative of more random texture. Some unrecrystallized regions are observed in (b). These regions have a sub-grain structure comprising of low angle boundaries. Thus, dynamic recrystallization during extrusion is not complete in the case of more dilute alloys (which effectively have a smaller amount of *i*-phase) when extruded at lower temperatures. Texture is stronger in more dilute alloys, as observed from the maximum values of texture intensity 3.13 and 3.71 in figures 15(c) and (d), respectively.

**Figure 16.**
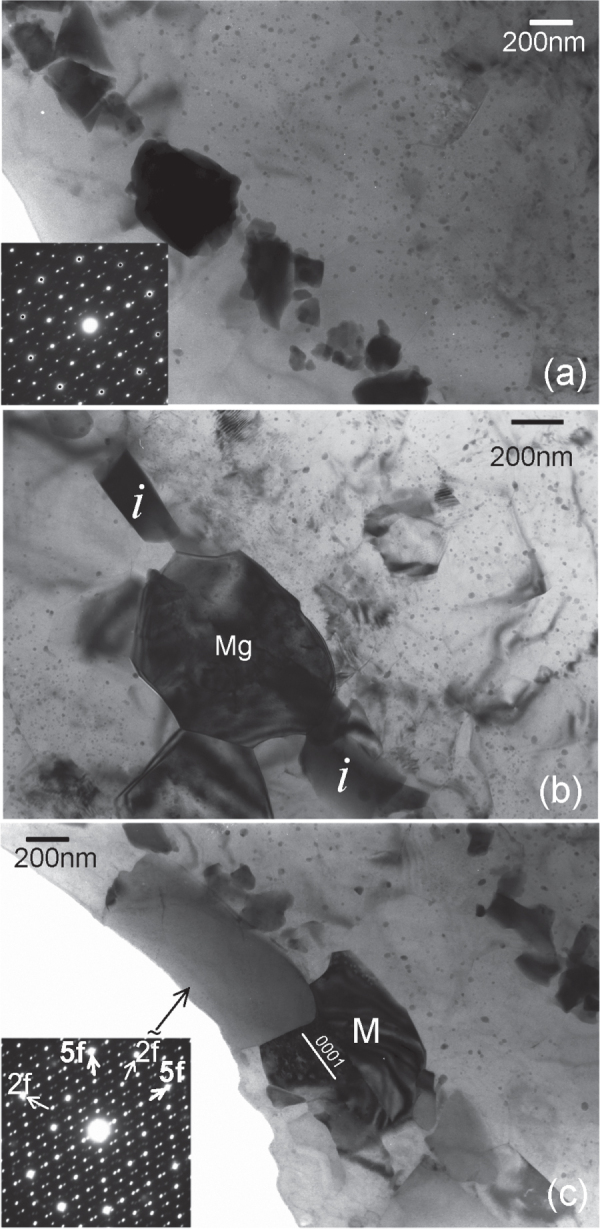
Bright field TEM micrographs from Mg-6Zn-1Y alloy chill cast and extruded at 250 °C. (a) *i*-phase particles dispersed along the extrusion direction. The inset shows a fivefold diffraction pattern from one of the particles. A ring of spots showing the fivefold symmetry are marked by dots. (b) Matrix grains in between *i*-phase particles marked *‘i’*. (c) An *i*-phase particle marked by an arrow and its inset diffraction pattern along a twofold zone axis. An adjacent matrix grain in dark contrast is marked ‘M.’ This matrix grain is along a 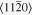 zone axis. Fine nano-sized precipitates are observed in all the micrographs.

**Figure 17.**
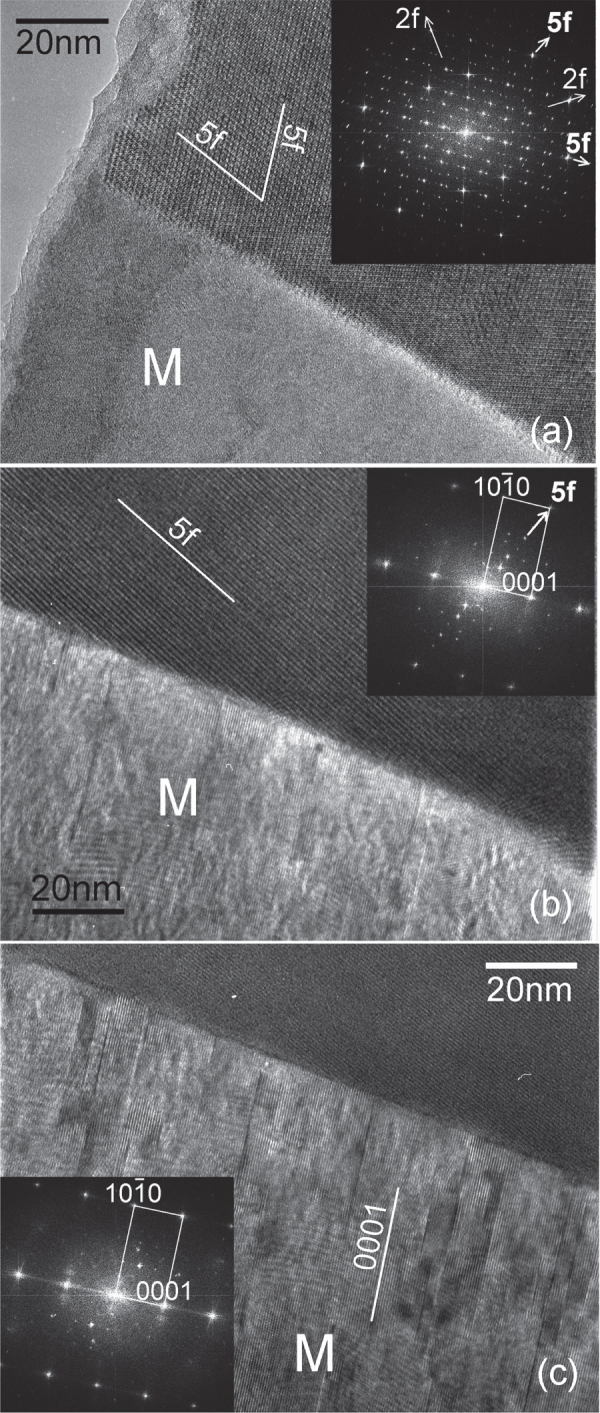
High resolution micrographs showing the interface between the *i*-phase and the matrix grain M in figure 16(c). (a) *i*-phase grain in a twofold zone axis orientation. The inset shows an FFT pattern, on which the twofold (2f) and fivefold (5f) reciprocal directions are marked. (b) A tilt away brings the grain M into a 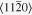 zone axis. One of the fivefold planes of the *i*-phase is still parallel to the beam, such that 5f 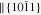 prismatic plane (as observed in the inset FFT pattern). (c) Stacking faults and slip are observed on basal planes in grain M, giving rise to streaking along 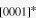 reciprocal direction in the inset FFT.

Some detailed microstructures are shown in figure 16, which are from the Mg-6Zn-1Y alloy extruded at 250 °C. Figure 16(a) shows *i*-phase particles dispersed along the extrusion direction. These particles are in the size range of 50 to 500 nm. Electron diffraction from a particle in the center oriented along a fivefold symmetry zone axis is inset. The poor quality of the diffraction and spots split in one direction indicates a high number of lattice defects and possible planar faults. This can result from faster solidification and mechanical stress from the extrusion process. Two matrix grains in between *i*-phase particles in figure 16(b) show very sharp boundaries. A matrix grain marked ‘M’ on the interface with an *i*-phase particle is shown in figure 16(c). The *i*-phase grain is close to a twofold zone axis orientation, whose diffraction pattern is inset. The grain M is in the 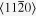 zone axis orientation, in which the orientation of the basal plane is indicated. The orientation of the basal plane in this grain is in approximately the same direction as the row of large *i*-phase particles, which are along the extrusion direction. Grains with basal planes aligned along the extrusion direction is in accordance with the basal texture of the extruded alloy. Details of the interface between this grain and the *i*-phase particle are shown by lattice images in figure 17.

In figure 17(a), the *i*-phase is in a twofold zone axis orientation (FFT pattern is inset). The interface with M is planar. Tilting away from this zone axis brings the grain M into a 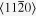 zone axis, as shown in figure 17(b), while one of the fivefold planes still remains parallel to the observation direction, so that 5f 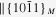. In figure 17(c) the grain M is exactly in the 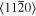 zone axis. It can be observed that the interface is very sharp, and nearly parallel to a prismatic plane. Stacking faults and basal slip are observed in grain M. However, these are not piled up on the interface.

The dynamically precipitated very fine precipitation of 10–15 nm size as observed in figures 14(d) and 16, provide precipitation strengthening, and also pin grain boundaries, preventing grain growth. As compared to the precipitate spacing *λ* on the slip plane for rod-like precipitates given by equation (2), the spacing of round precipitates is related to precipitate diameter *d* and volume fraction of precipitates *f* as

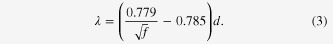
Thus for the same volume fraction *f* of precipitates the *λ* values will be smaller for spherical particles than rods, and strengthening will be larger.

The Mg-6Zn-1Y alloy was found to have supersaturation even after extrusion, which gave rise to an ageing response [18, 19]. Two ageing peaks were observed. One at 17 h was related to precipitation of rod-like 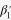 precipitates. A second peak at 48 h likely involved yttrium diffusion, after which no significant overageing occurred.

**Figure 18.**
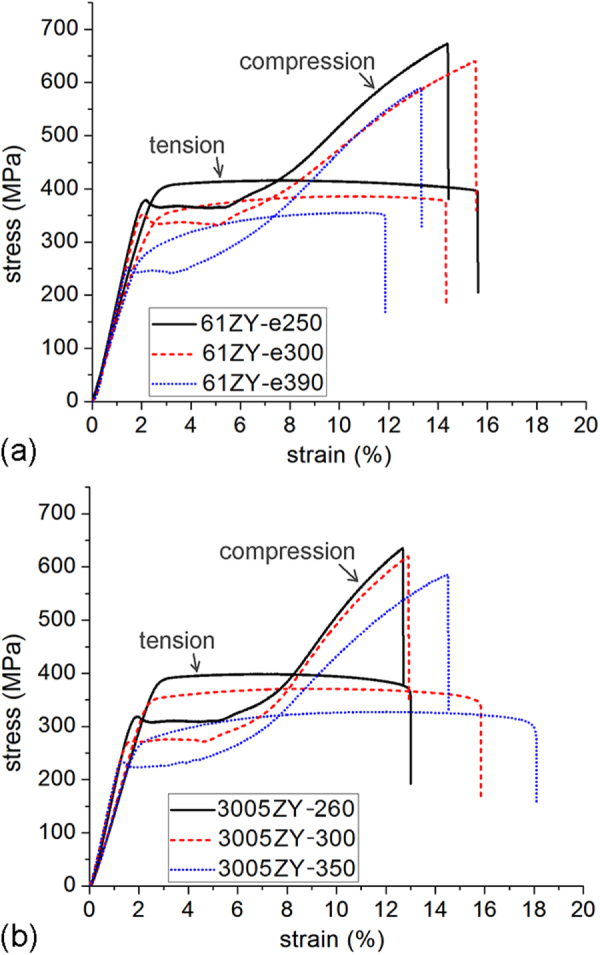
Engineering stress-strain plots for tensile and compression tests of alloys (a) Mg-6at%Zn-1at%Y and (b) Mg-3at%Zn-0.5at%Y, each extruded at three different temperatures.

Figure 18 shows the stress–strain curves of mechanical testing. Tensile and compressive yield strength are also listed in table 1. The stress–strain curves clearly show that with the decrease in grain size, there is increase in the ultimate tensile strength, the compressive strength and the tensile and compressive yield strength. In the case of 61ZY alloy, there is also an increase in the total elongation to failure with decreasing grain size. This may be a result of the weaker texture in the extruded 61ZY alloy. The textures were confirmed to be weaker in the 61ZY alloy than in the 305ZY alloy [52]. After an ageing treatment at 150 °C for 48 h, the tensile ductility of coarser grained alloys increased while that of the finer grained alloys decreased, as observed in table 1.

## 5. The role of the *i*-phase in strengthening

**Figure 19.**
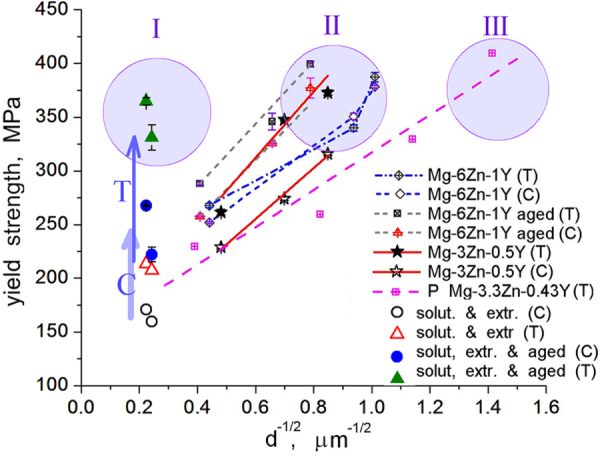
Hall–Petch plot of high strength Mg–Zn–Y alloys described here. ‘T’ represents yield strength in tension and ‘C’ represents compression. Data ‘P’ of powder metallurgy alloy is from [17]. Three regions of grain size-strength are delineated as I, II and III. ’Solut. & extr.’ stands for solutionized and extruded Mg-6at%Zn-1at%Y alloy.

Thus, by tailoring the microstructure of the Mg–Zn–Y alloys tensile yield strengths of over 350 MPa were achieved in a range of grain sizes, from over 20 *μ*m down to submicron level. This is illustrated in the Hall–Petch plot of figure 19. In case of the large grain size, a stable grain microstructure was created by precipitation of *i*-phase. The alloy was then strengthened by very fine precipitation of 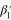 precipitates. Three regions I, II and III are marked in this Hall–Petch plot. In region I very high strength was achieved by very fine and dense precipitation of 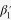 through a high temperature processing. In region II, ultra-high strength is achieved by a combination of ultra-fine grain size and nano-sized precipitation. Such a microstructure is obtained through a simple processing involving *i*-phase. The strengthening in region III, in which ultra-high strength is achieved by a powder metallurgy route, is dominated by fine grain size [17].

In ultra-high strength alloys with tensile yield strength of about 400 MPa and ductility of over 12%, the strengthening is mainly due to the ultra-fine grain size. Such small grain size is obtained in the alloys by rapid dynamic recrystallization due to the *i*-phase. Nano-size precipitates formed from the supersaturated matrix by dynamic precipitation help to pin the grain boundaries and retain a very fine grain structure [20]. It has been shown that *i*-phase particles strongly pin down grain boundaries [9].

**Figure 20.**
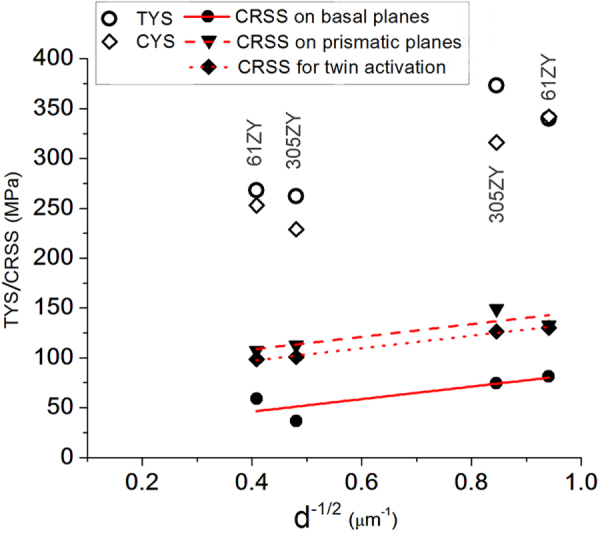
CRSS on basal and prismatic planes calculated from tensile yield strength, and for {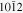} twinning calculated from compressive yield strength, for Mg-6Zn-1Y (61ZY) alloy extruded at 300 °C and 390 °C and Mg-3Zn-0.5Y (305ZY) alloy extruded at 260 °C and 350 °C.

From the grain orientation data of the extruded samples by EBSD technique (section 4.2, figure 15) the Schmidt factor 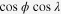 was calculated for basal and prismatic slips, where *ϕ* is the angle between the normal of the slip plane and applied stress direction, and *λ* is the angle between the slip direction and the applied stress. Yield strength was multiplied by the Schmidt factor to obtain critically resolved shear stress (CRSS) on basal and prismatic slip planes in tension and for {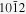} twinning in compression. These are plotted in figure 20 for four alloys, two each of two compositions extruded at two different temperatures. It is observed that the CRSS points for any particular type of slip or twin make a linear plot, independent of alloy composition and extrusion temperature, i.e., independent of the yield strength. Thus, strength and ductility of the alloys is strongly dependent on texture. Thus, *i*-phase dispersion produces a texture which balances high strength with ductility.

## 6. Conclusions

Microstructural studies involving icosahedral 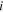-quasicrystalline and related phases in Mg–Zn–Y alloys, and the effect of processing on strength and ductility have been studied. The *i*-phase formed dendritically by solidification can be dispersed in the matrix by wrought processing such as rolling and extrusion, which also refines the matrix grain size. The *i*-phase can also be dispersed by dissolution and reprecipitation at high temperatures, but requires heterogeneous sites for nucleation. Precipitation of 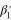 occurs in these alloys, whose structure is composed of related phases Mg_4_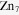 and 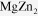, both closely related to *i*-phase in structure.

A high temperature annealing at the eutectic temperature in which *i*-phase is a eutectic phase, resulted in partial dissolution of the *i*-phase and transformation to a pre-eutectic hexagonal H phase. Elemental yttrium was also detected. Nano-sized *i*-phase particles nucleated on the grain boundaries and the matrix at lower temperatures. Thermal studies suggest three independent nucleation sites. Very fine precipitation of 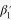 occurred on ageing at low temperatures. This processing resulted in very high tensile strengths of 370 MPa in spite of relatively large grain sizes of about 20 *μ*m.

Extrusion of chill cast Mg–Zn–Y alloys, which were partly supersaturated in solute elements, produced ultra-fine sub-micron grain size, and a fine distribution of nano-sized precipitates (∼15 nm) in the matrix. These alloys showed ultra-high strengths of 400 MPa with ductility of 12 to 16%. The presence of *i*-phase stimulated the recrystallization process during extrusion to produce ultra-fine grains, and at the same time moderated the texture to retain ductility and isotropy of deformation.

In final conclusion, by applying various processing routes based on phase transformations between *i*-phase and related phases, we have been able to get very high mechanical properties over a range of microstructures, from coarse grain size to ultra-fine. These alloys are unmatched in their combination of strength and ductility.
